# To Determine if Patients on Birkdale Ward Are Assessed for Risk of Venous Thromboembolism in Compliance With the Trust’s Standard Operating Procedure

**DOI:** 10.1192/bjo.2025.10567

**Published:** 2025-06-20

**Authors:** Akshita Dandawate

**Affiliations:** Mersey Care NHS Trust, Liverpool, United Kingdom

## Abstract

**Aims:** In August 2023 the Trust-wide standard operating procedure (SOP) titled ‘Prevention and Management of Venous Thromboembolism (VTE) for inpatients within Mersey care foundation trust (MCFT)’ was developed. The purpose for this was to explain the procedure to be followed for assessing and managing VTE in inpatient (IP) services as it is a common and potentially preventable problem. NICE Guidance recommends that all service users admitted to any hospital should receive a VTE risk assessment and prophylaxis where appropriate.

In secure settings, catatonia, use of supportive holds and sedation can be associated with reduced mobility and increased risk of VTE. Therefore the policy was updated to allow for use in Secure Divisions within MCFT as well.

The aims of this audit was to: critique the audit to ensure it is fit for purpose for trust-wide use and identify any shortfalls in the practice and if required to rectify these.

**Methods:** A VTE audit tool was initially designed on Trust AMAT software for Mental Health IP wards to complete, this was later adapted to reflect Secure IP. The tool assessed whether VTE was assessed appropriately during the total time of their admission as well as during the trigger points set out in the SOP.

All patients in one team on Birkdale ward were included: this was 8 in total.

**Results:** The overall compliance level was 0% which is extremely poor. 68% of patients were appropriately assessed during all trigger points in their admission however 38% were not and required an up-to-date VTE assessment.

**Conclusion:** As part of critiquing the audit tool, as mentioned before changes were made to questions in order to reflect the long-term admissions that patients can experience in secure settings. Due to this a time frame for data collection needed to be defined as using the total admission length was not viable.

In order to improve practice, all patients who required an up to date VTE assessment received one and the SOP was disseminated to the ward staff to increase awareness of what current practice is meant to be.

However many concerns were raised about applicability and effe ctiveness of the current SOP, both in acute and secure IP settings. Therefore following a discussion with the trust-wide team involved, a decision was made to amend the SOP. Following this, IP services will need to be re-audited to see if they are complaint with the new version.

